# A Case of Rosai–Dorfman Disease Complicated by Bone Metastasis From Prostate Cancer

**DOI:** 10.7759/cureus.82472

**Published:** 2025-04-17

**Authors:** Qiongying Chen, Yueqiao Zhao, Ke Zhou

**Affiliations:** 1 Radiology Department, Zigong Third People's Hospital, Zigong, CHN

**Keywords:** bone metastases, myeloproliferative disorder, palliative care, prostate cancer, rosai–dorfman disease

## Abstract

Rosai-Dorfman disease (RDD) is a rare myeloproliferative disorder of histiocytes. The cause of the disease remains unclear. RDD mainly affects lymph nodes and has many extranodal manifestations, including the skin, lungs, eyes, and gastrointestinal tract. This report details a diagnostically challenging case where retroperitoneal lymphadenopathy initially suspected as metastatic prostate adenocarcinoma was pathologically confirmed as RDD. The concurrent occurrence of these two distinct pathologies underscores the importance of histopathological verification in atypical presentations, particularly for guiding precision oncology management.

## Introduction

Histiocytic disorders originate from aberrant macrophage-monocyte differentiation, with the 2016 revised classification system delineating five molecular subgroups (L/C/R/M/H) based on MAPK pathway genetic alterations [1,2]. Classified under group R, Rosai-Dorfman disease (RDD) demonstrates polymorphic clinical manifestations ranging from isolated nodal enlargement to multiorgan infiltration. While cervical lymphadenopathy with constitutional symptoms (fever, elevated ESR, and weight loss) constitutes the classic phenotype [3], the present case exemplifies an atypical retroperitoneal presentation coexisting with metastatic prostate cancer, a clinical scenario scarcely documented in the existing literature.

## Case presentation

A 70-year-old male patient was admitted to a Grade III Class A public hospital in Sichuan Province, China, in November 2024 with worsening hypertension accompanied by lower limb swelling for >10 days. The patient had a history of hypertension for >10 years.

On physical examination, the patient had a blood pressure of 139/89 mmHg, was conscious, had a slightly anemic appearance, and his jugular vein was slightly distended. The patient had erythematous skin lesions of variable size (ranging from small to large) on both lower extremities. Some lesions had coalesced to form plaque-like formations. The affected areas demonstrated blanching upon pressure and exhibited elevated local skin temperature. Tumor marker levels were as follows: total prostate-specific antigen, >100.00 ng/mL (normal reference range: 0-4 ng/mL), and cancer antigen 125, 73.48 U/mL (normal reference range: 0-35 U/mL).

**Figure 1:**
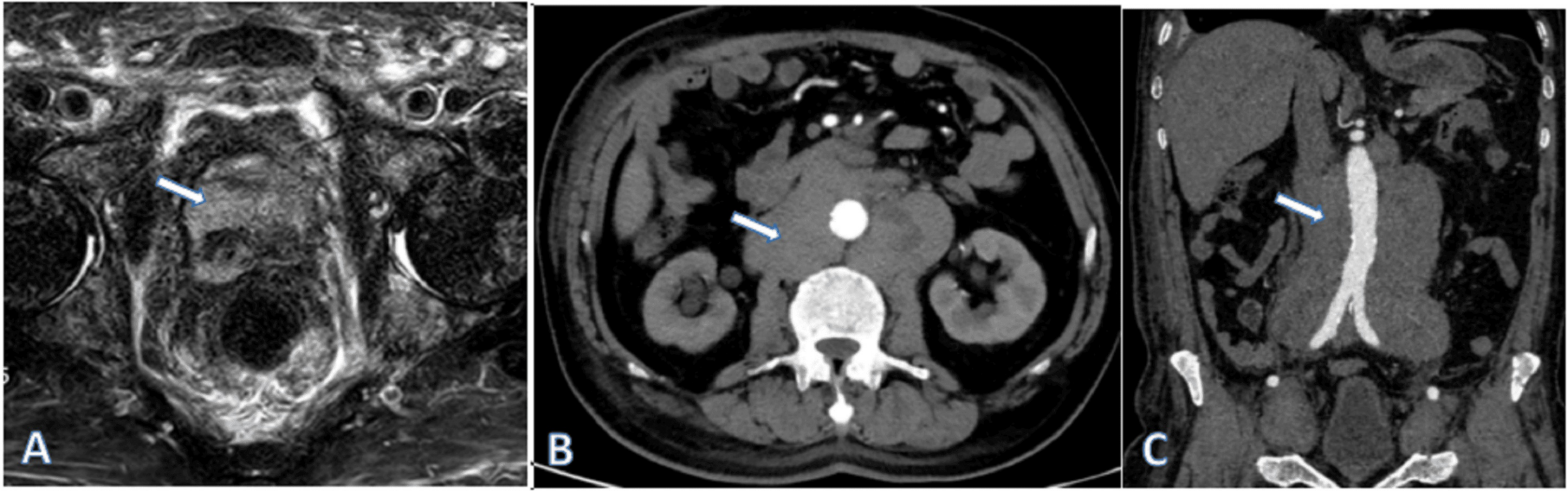
Prostate MRI and abdominal CT enhanced scan images. (A) MRI showing abnormal signals in the prostate. (B and C) Enhanced CT showing multiple enlarged lymph nodes around the abdominal aorta with uneven enhancement MRI: magnetic resonance imaging, CT: computed tomography

Pelvic MRI enhancement after admission revealed multiple abnormal signals in the prostate and multiple abnormal signal nodules in the pelvis and spine. Therefore, the radiographic preliminary diagnosis was prostate cancer (PI-RADS 5) with bone metastases (Figure 1A). Enhanced CT of the abdomen revealed multiple retroperitoneally enlarged lymph nodes with uneven enhancement. Hence, lymphoma was suspected (Figure 1B-1C).

**Figure 2:**
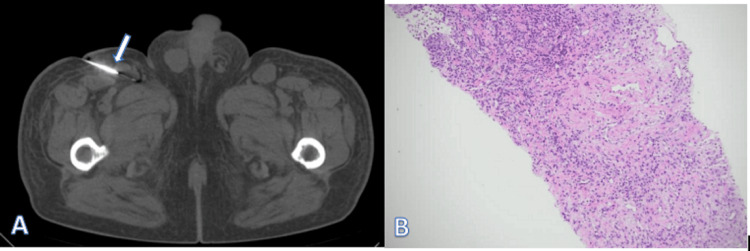
Right inguinal lymph node puncture and pathological image. (A) Right inguinal lymph node puncture. (B) Pathological results after lymph node puncture showing lymphoproliferative disease combined with immunohistochemically supported RDD RDD: Rosai-Dorfman disease

**Figure 3:**
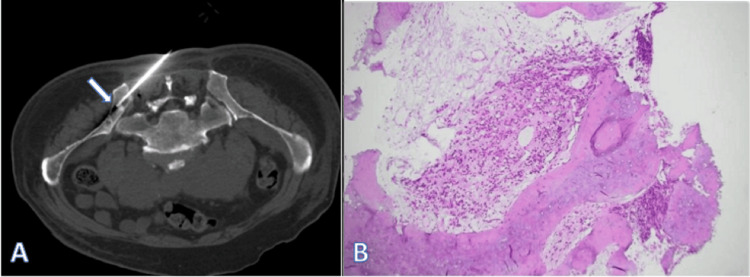
Iliac crest puncture and pathological image. (A) Pelvic bone puncture site. (B) Pathological findings showing that bone metastases tended to be of prostate origin (H&E ×100) H&E: hematoxylin and eosin

The patient underwent CT-guided right inguinal lymph node aspiration (Figure 2A) and CT-guided bone biopsy (Figure 3A) on November 22, 2024.

Lymph node biopsy pathological examination showed lymphoproliferative disease, with immunohistochemistry findings supporting the diagnosis of RDD (Figure 2B). Iliac bone marrow aspiration revealed metastatic cells consistent with prostatic carcinoma (Figure 3B).

Due to his advanced age, the patient was deemed unfit to tolerate additional treatment. Thus, the patient is currently maintained on palliative care treatment for symptomatic management using analgesics and other palliative treatment modalities.

## Discussion

RDD is a relatively rare disease, with an incidence of 1:200,000. Approximately 100 new cases are diagnosed annually in the United States [4]. RDD was initially described by Destombes [5]; subsequently, Azoury and Reed outlined and reported a classic case of RDD [6]. Later, Foucar et al. documented 34 cases and introduced the term “sinus histiocytosis with massive lymphadenopathy," later renamed RDD [7].

The etiology of RDD is unclear. However, RDD was previously classified as a benign histiocytic proliferative disease rather than a neoplastic process. Recent studies have shown that approximately one-third of patients with RDD harbor mutations in genes involved in the MAPK/ERK pathway, such as NRAS, KRAS, MAP2K1, and, rarely, BRAF, indicating a neoplastic process rather than a reactive disorder [8]. Consequently, RDD has been recently included in the fifth edition of the World Health Organization classification of myeloid and histiocytic/dendritic tumors [9].

RDD presents in two distinct forms: the first affects the lymph nodes with or without systemic symptoms and extranodal manifestations, whereas the second is exclusively cutaneous without systemic or nodal involvement [10].

RDD occurs in isolation or association with autoimmune or neoplastic diseases, including classical nodal and extranodal diseases, and may be sporadic or familial [11]. In the present case, RDD occurred concomitant with prostate cancer, supporting the hypothesis of a potential relationship between RDD and malignancy.

Patients with RDD often present with B symptoms (fever, night sweats, and weight loss). Approximately 43% of patients develop extranodal manifestations [12], predominantly affecting the skin, nasal cavity, or orbit. Other involved sites include the salivary glands, spleen, bone, and testes [13]. Typically, the lesions are located in different parts of the body, resulting in varying imaging findings. In 57% of cases, RDD was located in the nodal node, and imaging findings showed bilateral cervical, mediastinal, retroperitoneal, and inguinal lymph node enlargement with uneven enhancement on enhanced scans. Therefore, RDD occurring in lymph nodes should be differentiated from lymphoma and cancerous lymph node metastasis. RDD is a histiocytic dysplasia with multiple clinical manifestations that can occur at multiple sites, either in isolation or in combination with other diseases. It requires a comprehensive clinical, radiological, pathological, and molecular diagnostic approach.

Owing to the rarity of this disorder, no standard therapy has been established for patients with RDD based on prospective clinical trial data. Treatment strategies are based on the findings of retrospective case series, case reports, disease-registry analyses, and expert opinions. Only recently, the NCCN Clinical Practice Guidelines in Oncology, Histiocytic Neoplasms, version 2.2021, proposed diagnostic and treatment strategies for patients with RDD [14]. As 40% of patients with nodal/cutaneous involvement experience spontaneous remission, clinical observation or "watchful waiting" is considered necessary for asymptomatic or mildly symptomatic patients [15]. For symptomatic RDD in lymph nodes or other sites, targeted or combination therapy may improve the prognosis of patients with RDD.

## Conclusions

Intralymph node RDD occurring in the setting of malignant tumors is very rare, and the initial diagnosis is very difficult. Accordingly, diagnosis requires the full combination of clinical data and laboratory examinations. Accurate preoperative imaging diagnosis is very important for making accurate treatment decisions, as RDD needs to be distinguished from other diseases causing lymph node enlargement.
